# Optimization of Fermentation Conditions and Media for Production of Glucose Isomerase from *Bacillus megaterium* Using Response Surface Methodology

**DOI:** 10.1155/2018/6842843

**Published:** 2018-09-02

**Authors:** Hoang-Yen Thi Nguyen, Gia-Buu Tran

**Affiliations:** Institute of Biotechnology and Food-Technology, Industrial University of Ho Chi Minh City, 12 Nguyen Van Bao Street, Go Vap, Ho Chi Minh, Vietnam

## Abstract

Glucose isomerase is an enzyme widely used in food industry for producing high-fructose corn syrup. Many microbes, including *Bacillus megaterium*, have been found to be able to produce glucose isomerase. However, the number of studies of glucose isomerase production from *Bacillus megaterium* is limited. In this study, we establish the optimal medium components and culture conditions for *Bacillus megaterium* glucose isomerase production by evaluating the combined influence of multiple factors and different parameters via Plackett–Burman design and response surface methodology in Modde 5.0 software. The optimized conditions, which were experimentally confirmed as follows: D-xylose (1.116%), K_2_HPO_4_ (0.2%), MgSO_4_·7H_2_O (0.1%), yeast extract (1.161%), peptone (1%), pH 7.0, inoculum size 20% (w/v), shaking 120 rpm at 36.528°C for 48 hours, give rise to production of highest activity of glucose isomerase (0.274 ± 0.003 U/mg biomass). These results provide additional important information for future development of large-scale glucose isomerase production by *Bacillus megaterium.*

## 1. Introduction

High-fructose corn syrup (HFCS) is a mixture of glucose-fructose which is widely used as a low-calorie sweetener in food industry. Treatment with isomerase enzymes, such as glucose isomerase, provides a highly efficient and cost-saving method for production of HFCS. Glucose isomerase, also known as D-glucose isomerase and D-xylose isomerase, is the intracellular enzyme which catalyzes the isomerization of glucose to fructose and xylose to xylulose; therefore, it accelerates the sweetness of food product and plays an important role is HFCS production. In 1957, Marshall and Kooi for the first time reported that an enzyme existing in intact cell or sonic extract of *Pseudomonas hydrophila*, lately named as glucose isomerase, could convert the D-glucose to D-fructose to produce the high-fructose corn syrup [1]. Furthermore, glucose isomerase was firstly applied for high-fructose corn syrup production in industrial scale by Clinton Corn Processing Co. in 1967 [2]. Since then, the utilization and demand of HFCS in food industry have noticeably increased for decades. Up to now, glucose isomerase is one of the most common enzymes in food industry due to its application in HFCS production [3]. Moreover, glucose isomerase is used for ethanol production, since glucose isomerase can also convert xylose into xylulose, which provides nutrient for saprophytic bacteria and supports biosynthesis of hemicellulose to produce bioethanol. On the market, commercialized glucose isomerase products are sold as immobilized enzymes and cells. Enzymatic activity of these products is regulated by a variety of factors such as the presence of metal cations, microbial sources, pH, and temperature. The presence of some divalent metal cations, such as Mg^2+^ and Co^2+^ or Mn^2+^, has been proved for enhancing glucose isomerase activity, whereas other metal cations including Ag^+^, Hg^2+^, Cu^2+^, Zn^2+^, Ni^2+^, and Ca^2+^ have been proved for decreasing enzymatic activity. Moreover, enzymatic activity of glucose isomerase also is inhibited by xylitol, arabitol, mannitol, and so on [4].

Several microbial sources have been used for production of glucose isomerase in laboratory scale, such as *Streptomyces* spp., *Arthrobacter* spp., *Clostridium thermosulfurogenes*, *Pseudomonas* spp., *Thermoanaerobacter* spp., *Thermoanaerobacterium* spp., *Bifidobacterium* spp., and *Bacillus* spp. [4, 5]. Most of commercialized glucose isomerases used in industrial scale are produced from *Streptomyces* spp., *Arthrobacter* spp., *Actinoplanes missouriensis*, and *Bacillus coagulans*. However, the number of studies using *Bacillus megaterium* for production of glucose isomerase in pilot and industrial scale is limited. *Bacillus megaterium*, a mesophilic bacterium, has been known as the important industrial microorganism for producing penicillin amidase, amylase, glucose dehydrogenase, and fungicidal and antiviral agents for a long time. In 1962, Takashima and Takabe firstly isolated glucose isomerase from *Bacillus megaterium*, which named as D-glucose-ketol-isomerase and exerted distinguish characteristics with other glucose isomerases. This enzyme was a NAD^+^-linked enzyme and showed a high specificity for glucose [6]. Furthermore, Mukesh-Kumar et al. determined the effect of individual fermentation factors such as pH, temperature, and cultivation time on the one-at-a-time method [7]. However, the combined influence of multiple factors of cultivation parameters, such as nutrient components (D-xylose, K_2_HPO_4_, MgSO_4_, peptone, and yeast extract) and cultivation conditions (rotation rate, pH, temperature, inoculum size, and cultivation time), on the biosynthesis of glucose isomerase in *Bacillus megaterium* has not been elucidated yet. In this study, we investigate the combined influence of multiple factor of cultivation parameters, such as nutrient composition and cultivation conditions, on production of glucose isomerase and optimize the main cultivation parameters for *Bacillus megaterium* to produce maximal glucose isomerase activity using the Plackett–Burman design and response surface methodology in Modde 5.0 software.

## 2. Materials and Methods

### 2.1. Chemicals

All reagents were obtained from HiMedia Laboratories Ltd. (Mumbai, India) in microbiological or analytical grade unless otherwise noted. Carbazole (Supelco 442506) and cysteine hydrochloride (C7880) were purchased from Sigma-Aldrich Ltd. (Missouri, USA).

### 2.2. Bacterial Strain, Growth Curve Measurement, Glucose Isomerase Production, and Extraction

The *Bacillus megaterium* strain used in this study was isolated from shrimp farming pond, identified, and deposited in bacterial collection of Laboratory of Biotransformation, University of Science, Vietnam National University Ho Chi Minh City.

Single colony of bacterial strain was grown overnight at 37°C in enriched medium (0.25% yeast extract, 0.5% peptone, 0.15% K_2_PO_4_, 0.5% D-xylose, 0.05% MgSO_4_·7H_2_O, pH 7). The turbidity of overnight culture was adjusted by adding the enriched medium to reach final OD_600_ = 0.5. Five milliliters of the overnight culture (OD_600_ = 0.5) was added in 200 mL of fresh enriched medium, and the growth curve was established by monitoring cell density (CFU/mL) every 2 hours for 40 hours via plate count method.

Twenty percent of stationary phase culture (22 hours after incubation) was inoculated into fresh induction medium (0.5% yeast extract, 1% peptone, 0.3% K_2_PO_4_, 1% D-xylose, 0.1% MgSO_4_·7H_2_O, pH 7.0) for producing glucose isomerase. The bacterial culture was incubated at 37°C with rotation rate 120 rpm for 48 hours. After incubation, the suspension was centrifuged and the supernatant was removed. The pellet was collected and the biomass weight (mg biomass) was determined. Then, the cells were dissolved by extraction buffer containing 0.9% NaCl, 0.1% lysozyme, and 1% toluene into final concentration 20 mg biomass/1.5 mL suspension. Cell debris was removed by centrifugation, and crude extract was used for further experiments.

### 2.3. Determination of Glucose Isomerase Activity

Glucose isomerase activity was determined using the previously reported method with some modification [7]. Briefly, 0.2 mL of cell suspension was added into 1.8 mL of the isomerization reaction mixture containing 0.5 mL of 0.2 M Na_3_PO_4_, 0.1 mL of 0.1 M MgSO_4_, 0.1 mL of 0.01 M CoCl_2_·6H_2_O, and 0.2 mL of 1.0 M glucose. The reaction mixture was incubated at 70°C for 1 hour, and then 2 mL of 0.5 M perchloric acid was successively added to stop the reaction. The amount of fructose in reaction mixture was measured using cysteine-carbazole-sulfuric acid method with a standard curve of fructose [8]. One unit of glucose isomerase activity was defined as the amount of enzyme that generates 1 µmole of fructose per minute under assay condition. The results were presented as U/mg biomass.

### 2.4. Determination of the Influence of Individual Components of Induction Medium on Glucose Isomerase Production

The effect of various nutrient components of medium on glucose isomerase production was assessed by growing bacterial culture in induction medium (0.5% yeast extract, 1% peptone, 0.3% K_2_PO_4_, 1% D-xylose, and 0.1% MgSO_4_·7H_2_O) at 37°C with rotation rate 120 rpm for 48 hours using single-factor experiments. For optimizing carbon source concentration, the induction medium was prepared with substitution of a variety of concentrations of D-xylose from 0 to 2.5% with 0.5% interval. The optimal concentration of dipotassium phosphate was determined by replacement with various concentrations of dipotassium phosphate (0, 0.1, 0.2, 0.3, 0.4, and 0.5%) in induction medium formula. The effect of different concentrations of MgSO_4_ (0, 0.05, 0.1, 0.15, 0.2, and 0.25%) was also analyzed. To investigate the effect of different concentrations of nitrogen source, the concentration of yeast extract of induction medium was changed from 0 to 1.25% at 0.25% interval. Furthermore, the effect of different concentrations of peptone (0, 0.5, 1, 1.5, 2, and 2.5%), the second nitrogen source, was also investigated. After that, the enzymatic activity assay was determined by the method mentioned in previous section.

### 2.5. Determination of the Influence of Individual Fermentation Conditions on Glucose Isomerase Production

The effect of fermentation factors on glucose isomerase production was determined by growing bacterial culture in induction medium (0.5% yeast extract, 1.0% peptone, 0.3% K_2_PO_4_, 1.0% D-xylose, and 0.1% MgSO_4_·7H_2_O) for 48 hours using single-factor experiments. To investigate the effect of pH on glucose isomerase production, pH of induction media was adjusted from 5.5 to 8.0 at 1.0 unit interval. A variety of bacterial inoculum percentages (5, 10, 15, 20, 25, and 30%) was inoculated into induction medium. The optimal agitation was selected by shaking the bacterial culture at a range of rotation rates (80, 100, 120, 140, 160, and 180 rpm). The effect of varying incubation temperatures was determined via incubation of bacterial culture at a range of temperatures from 33 to 44°C with 2°C interval. For all conditions, the enzymatic activity assay was determined by the method mentioned in previous section.

### 2.6. Optimization Fermentation Conditions and Medium Composition for Production of Glucose Isomerase

To optimize the fermentation conditions and medium composition for production of glucose isomerase, the conditions that had significant effects on glucose isomerase production were identified by the Plackett–Burman design. In this design, eight variables, such as the concentrations of D-xylose, MgSO_4_, K_2_HPO_4_, peptone, and yeast extract, as well as pH, bacterial inoculum percentage, temperature, and rotation rate, were selected for analysis by the Plackett–Burman design and the factors with a confidence level above 95% were employed in further optimization. The results were analyzed by response surface methodology to establish the response surface and contour plots for visualization of the relationship between the experimental variables and response and selection of the optimal variable.

### 2.7. Statistical Analysis

All experiments were replicated. Statistical analysis was performed using statistical R software (Lucent Technologies). Differences between means of different groups were analyzed using analysis of variance (ANOVA) and Fisher LSD test, and the criterion of statistical significance was set as *p* < 0.05. The data were presented as mean ± standard deviation.

## 3. Results and Discussions

### 3.1. Establishment of *Bacillus megaterium* Growth Curve in Enriched Medium

**Figure 1:**
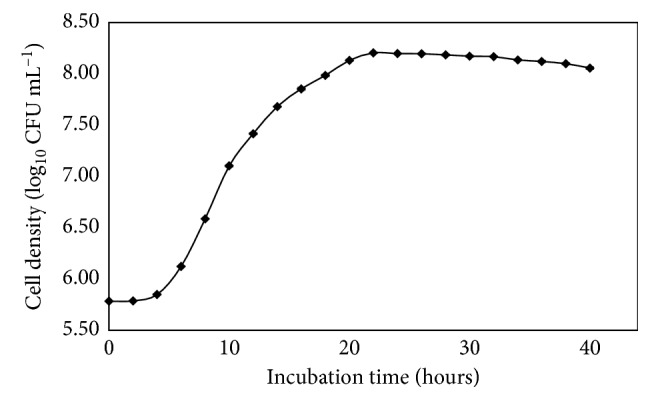
Growth curve of *Bacillus megaterium* in enriched medium. The bacteria reached the maximal value after 22 hours of inoculation in enriched medium. Therefore, we chose that time point (22 hours) for bacterial inoculation in induction medium to obtain the highest amount of viable bacteria.

In this study, we established the growth curve of *Bacillus megaterium* in enriched medium containing 0.25% yeast extract, 0.5% peptone, 0.15% K_2_PO_4_, 0.5% D-xylose, and 0.05% MgSO_4_·7H_2_O. After inoculation, bacteria density was steady during the first 6 hours, which implied bacteria had to adapt to new medium and the period from 0 hour to 6 hours was the lag phase. The next period from 8 hours to 22 hours, bacteria have exponentially grown and bacteria density dramatically increased, which implied this period was the exponential phase of the growth curve. After 22 hours, the bacteria density reached the maximal value and unchanged until 26 hours, which indicated that the period from 22 hours to 26 hours was stationary phase of the growth curve. After 28 hours, bacteria density was decreased, which suggested the death phase (Figure 1). In order to obtain the highest growth yield and shorten the lag phase in new media, we suggested that inoculation of bacteria in induction medium was performed at 22 hours after being inoculated in enriched medium.

### 3.2. Effect of Concentrations of Induction Medium Components on Glucose Isomerase Production

**Figure 2:**
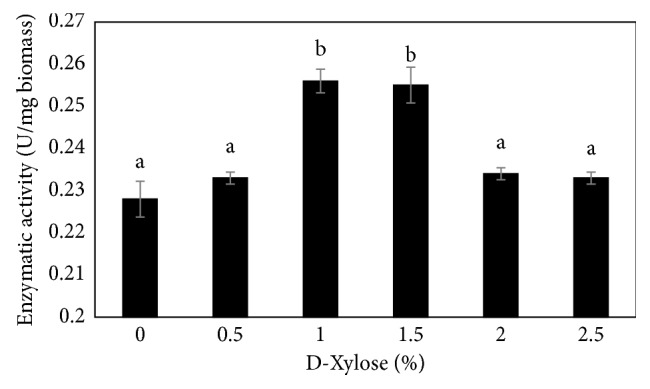
The effect of the concentration of D-xylose on glucose isomerase production. We found that the increase of concentration of D-xylose (1–1.5%) will result into improvement of bacterial growth and activation of glucose isomerase biosynthesis. However, the higher concentration of D-xylose (2–2.5%) could inhibit the bacterial growth, which decreased enzyme activity. Statistical analysis revealed no significant difference between 1% and 1.5% D-xylose on enzyme production; therefore, we chose 1% of D-xylose for further optimization experiments. a and b indicate the significant difference between groups.

As shown in Figure 2, the enzymatic activity was progressively increased from 0.228 to 0.255 U/mg biomass when the concentration of D-xylose increased from 0 to 1.5%. However, the concentration of D-xylose was higher (from 2.0 to 2.5%), and the enzyme activity was reduced from 0.255 to 0.233 U/mg biomass. D-Xylose is the main carbon source of bacteria which plays an important role for bacterial growth. Moreover, D-xylose is also the inductive substrate for converting from glucose to fructose by glucose isomerase. Therefore, the increase of concentration of D-xylose (1.0–1.5%) will result into improvement of bacterial growth and activation of glucose isomerase biosynthesis. However, higher concentration of D-xylose (2.0–2.5%) could inhibit the bacterial growth and decrease enzyme activity (Figure 2). In previous study, Prabhakar and Raju observed that medium supplemented with 1% of D-xylose in medium gave rise to the maximal activity of glucose isomerase in *Arthrobacter spp.* whereas the higher concentration of D-xylose (2%) dramatically reduced enzymatic activity and bacterial biomass, which is consistent with our result [9]. Statistical analysis revealed that there is no significant difference between 1% D-xylose and 1.5% D-xylose (0.256 ± 0.003 versus 0.255 ± 0.004 U/mg biomass). For that reason, we chose 1% D-xylose for induction of glucose isomerase production.

**Figure 3:**
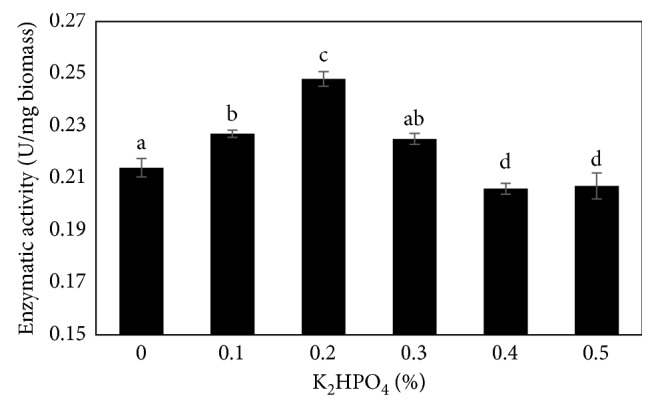
The effect of the concentration of K_2_HPO_4_ on glucose isomerase production. Enzyme activity was significantly increased after supplement with 0.2% of K_2_HPO_4_ as compared to medium without K_2_HPO_4_. However, we observed a decline of enzyme activity while the concentration of K_2_HPO_4_ was adjusted from 0.3 to 0.5% (0.225 ± 0.002 and 0.207 ± 0.005 U/mg biomass, accordingly, *p* < 0.05). We chose the optimal concentration of K_2_HPO_4_ is 0.2%. a, b, c, and d indicate the significant difference between groups.

The effect of K_2_HPO_4_ concentration on glucose isomerase production was also assessed. Briefly, enzymatic activity was significantly increased after supplement with 0.2% of K_2_HPO_4_ as compared to the one without K_2_HPO_4_ (0.248 ± 0.003 versus 0.219 ± 0.004 U/mg biomass, resp., *p* < 0.05). However, we observed a decline of enzymatic activity, while the concentration of K_2_HPO_4_ was adjusted from 0.3 to 0.5% (0.225 ± 0.002 and 0.207 ± 0.005 U/mg biomass, accordingly, *p* < 0.05). Taken together, we determined that the optimal concentration of K_2_HPO_4_ is 0.2% (Figure 3). Phosphate is the main component of ATP, nucleotide, and ribonucleotide; thereby, it plays an important role in energy production, nucleic acid, and protein biosynthesis. Consequently, the deficiency of phosphate in induction media, such as medium without K_2_HPO_4_ or medium containing low concentration of K_2_HPO_4_, resulted in a decrease of glucose isomerase activity. On the contrary, the excess of phosphate concentration would cause a decrease of protein synthesis, which was reported in previous study [10]. The authors elucidated that the accumulation of inorganic phosphate would hinder protein synthesis via reducing concentration of free magnesium ion. Note that magnesium ion not only plays important role in protein synthesis but also is the activator of glucose isomerase. Therefore, increase of K_2_HPO_4_ concentration has an inhibitory effect on glucose isomerase activity. Moreover, Gersch et al. also observed an inhibition effect of phosphate on biosynthesis of turimycin, the secondary metabolite of *Streptomyces hygroscopicus* [11].

**Figure 4:**
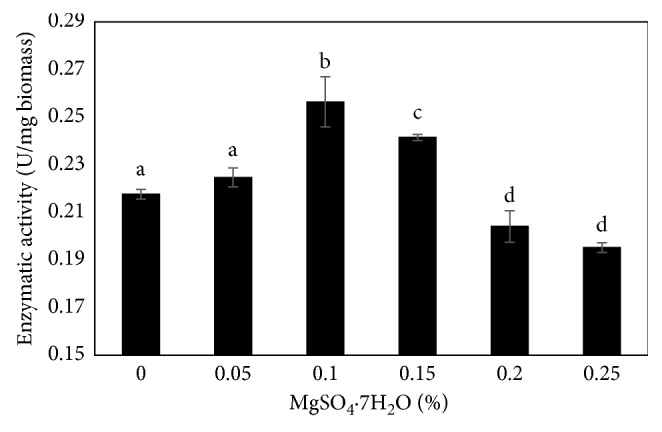
The effect of the concentration of MgSO_4_·7H_2_O on glucose isomerase production. The concentration of MgSO_4_·7H_2_O was increased (0-0.1%) along with enhancement of enzyme activity. We observed that the higher concentration of MgSO_4_·7H_2_O (0.15–0.25%) resulted in a decline of enzyme activity. We chose 0.1% of MgSO_4_·7H_2_O to further optimization experiments. a, b, c, and d indicate the significant difference between groups.

MgSO_4_·7H_2_O provides magnesium ion, which is required for bacterial growth and activation of glucose isomerase activity. We found that the enzymatic activity was increased along with the increase of MgSO_4_·7H_2_O concentration from 0 to 0.1% (0.223 ± 0.002 and 0.264 ± 0.011 U/mg biomass, resp., *p* < 0.05). Note that the higher concentration of MgSO_4_·7H_2_O (0.15–0.25%) resulted in a decline of enzymatic activity from 0.248 ± 0.001 to 0.199 ± 0.002 U/mg biomass (Figure 4). From these data, we chose the optimal concentration of MgSO_4_·7H_2_O is 0.1%. These data were similar with the results reported by Chen et al., in which they observed that the optimal concentration of MgSO_4_·7H_2_O for glucose isomerase production of *Streptomyces flavogriseust* was 0.1% with the highest enzyme activity (1.93 U/mg protein); either the lower concentration (0.03%) or the higher concentration (0.5%) resulted in a decline of enzyme activity [12].

**Figure 5:**
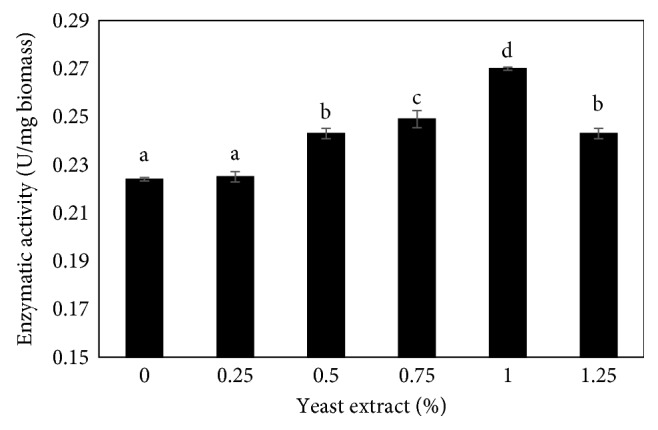
The effect of the concentration of yeast extract on glucose isomerase production. Yeast extract provides nitrogen for production of glucose isomerase but high concentration will inhibit the bacterial growth. In this study, we determined the optimal concentration of yeast extract is 1%. Addition of yeast extract (0–1%) in induction medium resulted in an increase of enzyme activity, but yeast extract also exhibited the inhibitory effect in high level (1.25%). a, b, c, and d indicate the significant difference between groups.

Nitrogen requirement of microorganisms are diverse; therefore, the nitrogen source and their optimal concentration are also varied among microorganisms. In this study, the importance of yeast extract and peptone on glucose isomerase production was investigated. We observed that yeast extract concentration adjusted from 0 to 1% accompanied with an increase of enzyme activity from 0.224 ± 0.001 to 0.270 ± 0.001 U/mg biomass (*p* < 0.05). Yeast extract is the main nitrogen source for bacterial growth and protein synthesis; thereby, the increase of yeast extract accelerates glucose isomerase production. Some authors suggested that the optimal concentration of yeast extract for glucose isomerase production was in a range from 0.5-1%, which is identical with our results [9, 13]. On the other hand, yeast extract concentration reached the maximum value (1.25%) and enzyme activity reduced to 0.243 ± 0.001 U/mg biomass (Figure 5). From these data, we chose 1% of yeast extract as the optimal concentration of yeast extract for producing glucose isomerase.

**Figure 6:**
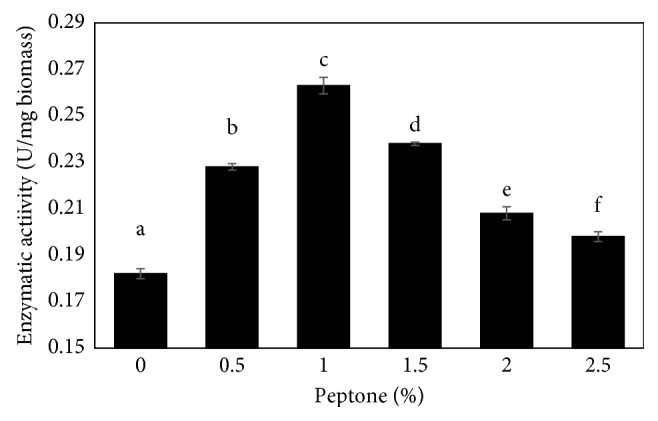
The effect of the concentration of peptone on glucose isomerase production. Peptone is the second nitrogen source for bacterial growth and enzyme synthesis; therefore, the concentration of peptone may affect the glucose isomerase activity. In this study, the concentration of peptone was increased from 0 to 1% along with an elevation of enzyme activity from 0.182 ± 0.002 to 0.263 ± 0.004 U/mg biomass (*p* < 0.05). Then, the enzyme activity decreased along with the higher increase of peptone concentration (1.5–2.5%). Therefore, we chose 1% peptone as the optimal concentration to produce glucose isomerase.

Peptone is one of the common organic nitrogen sources for microorganisms and plays the key role in glucose isomerase production in some microorganisms including *Lactobacillus bifermentans* and *Streptomyces thermonitrificans* [14, 15]. As shown in Figure 6, when we increased the concentration of peptone from 0 to 1%, enzymatic activity elevated from 0.182 ± 0.002 to 0.263 ± 0.004 U/mg biomass (*p* < 0.05). The enzymatic activity then decreased along with the higher increase of peptone concentration (1.5–2.5%). Therefore, we chose 1% peptone as the optimal concentration to produce glucose isomerase. This finding was consistent with the results of previous research [16], in which the authors reported the optimal concentration of nitrogen source for *Streptomyces spp.* to produce glucose isomerase was 1% peptone. Taken together, the optimal induction medium for glucose isomerase production from *Bacillus megaterium* contains 1% D-xylose, 0.2% K_2_HPO_4_, 0.1% MgSO_4_·7H_2_O, 1% yeast extract, and 1% peptone.

### 3.3. Effect of Fermentation Conditions on Glucose Isomerase Production

**Figure 7:**
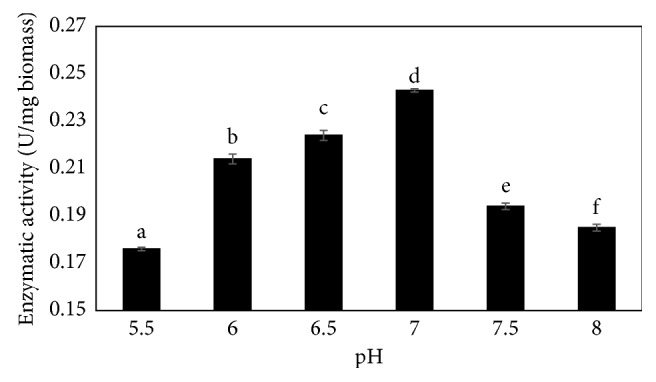
The effect of pH on glucose isomerase production. In this study, we observed an increase of enzyme activity (0.176 ± 0.01 to 0.243 ± 0.01 U/mg biomass) along with an increase of pH value of induction medium (pH 5.5–pH 7.0). However, when pH increased from 7.5 to 8.0, we observed a decline of enzyme activity (from 0.194 ± 0.01 to 0.185 ± 0.01 U/mg biomass, resp.). From these data, we chose optimal pH value for glucose isomerase production was 7.0. a, b, c, d, e, and f indicate the significant difference between groups.

The effect of pH on glucose isomerase production is presented in Figure 7. When the pH of induction medium increased from 5.5 to 7.0, the enzymatic activity increased from 0.176 ± 0.01 to 0.243 ± 0.01 U/mg biomass (*p* < 0.05). However, the higher pH range (pH 7.5–pH 8.0) would inhibit the enzyme activity (from 0.194 ± 0.001 to 0.185 ± 0.001 U/mg biomass, resp.). The optimal pH value (pH 7.0) to obtain maximal activity of glucose isomerase is in the range for bacterial growth (pH 5.7–pH 7.0). Moreover, optimal pH value is neutral pH, which facilitates glucose isomerase production in industrial scale because pH adjustment does not need to perform. In previous report, Yassien and Jiman-Fatani suggested that pH 7.0 was optimal for microbial growth and glucose isomerase production of *Streptomyces albaduncus*, which was consistent with our results [17]. On the contrary, some authors determined the optimal pH for *Bacillus megaterium* BPTK5 is 6.0 [7]. The difference in optimal pH values of *Bacillus megaterium* between this study and Mukesh-Kumar research may be accounted for by the different isolated sources. Note that the *Bacillus megaterium* strain used in the present study was isolated from shrimp farming pool in Vietnam, whereas *Bacillus megaterium* BPTK5 strain was isolated from cassava waste.

**Figure 8:**
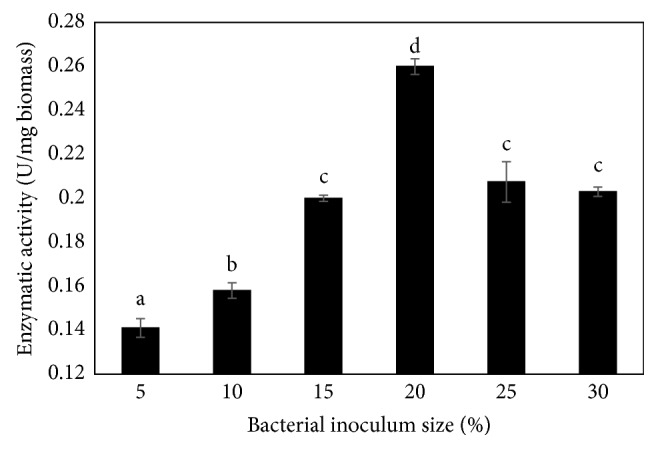
The effect of bacterial inoculum size on glucose isomerase production. In this study, we determined the optimal bacterial inoculum size is 20%. Addition of bacteria (5–20%) in induction medium resulted an increase of enzyme activity, but bacterial inoculum size also exhibited the inhibitory effect at the higher levels (25–30%). a, b, c, and d indicate the significant difference between groups.

The influence of bacterial inoculum size on production of glucose isomerase was also investigated (Figure 8). We observed an increase of enzyme activity when the bacterial inoculum size elevated from 5% to 20% (0.141 ± 0.004 versus 0.260 ± 0.004 U/mg biomass, resp., *p* < 0.05). However, the higher inoculum size (25–30%) resulted in a decrease of enzymatic activity as compared to medium inoculum size (20%). Therefore, we chose the optimal bacterial inoculum size was 20%. When the amount of bacterial inoculum is too low, bacteria were slowly grown and enzymatic activity is low. The increase of inoculum size will increase the interaction between substrate, nutrient, and bacteria, which in turn enhance bacterial metabolism, protein synthesis, and enzyme activity. However, the high amount inoculum size leads to nutrient and substrate competition as well as the decrease of enzyme activity.

**Figure 9:**
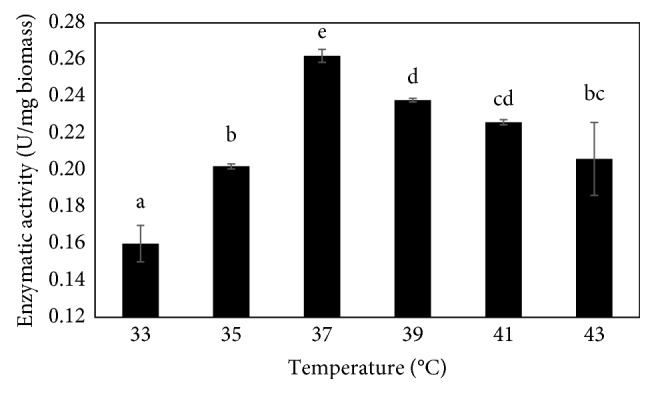
The effect of temperature on glucose isomerase production. Enzyme activity was increased along with an elevation of temperature from 33°C to 37°C (*p* < 0.05). When the temperature increased over optimal temperature (39°C–43°C), the enzyme activity dramatically decreased from 0.238 ± 0.001 to 0.206 ± 0.020 U/mg biomass (*p* < 0.05). From these data, we chose 37°C as the optimal temperature for glucose isomerase production. a, b, c, d, and e indicate the significant difference between groups.

Temperature is an important factor that affects the bacterial growth and enzyme biosynthesis. Each strain of bacteria adapts to an optimal range of temperature in which it promptly grows and synthesizes protein. If bacteria are cultured at the higher temperature, they will be weakened and reduce enzyme production. As shown in Figure 9, enzymatic activity was increased along with an elevation of temperature from 33°C to 37°C (0.160 ± 0.010 versus 0.262 ± 0.004 U/mg biomass, accordingly, *p* < 0.05). When the temperature increased over optimal temperature (39°C–43°C), the enzyme activity dramatically decreased from 0.238 ± 0.001 to 0.206 ± 0.020 U/mg biomass (*p* < 0.05). From these data, we chose 37°C as the optimal temperature for glucose isomerase production.

**Figure 10:**
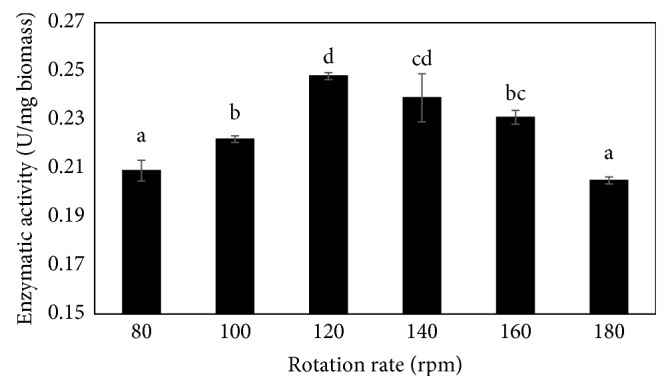
The effect of rotation rate on glucose isomerase production. Rotation rate has close relation with dissolved oxygen and nutrient diffusion in media. When the rotation rate increased from 80 to 120 rpm, the enzyme activity significantly increased (0.209 ± 0.004 and 0.248 ± 0.001 U/mg biomass, resp., *p* < 0.05). We observed that the faster rotation rate (140–180 rpm) reduced the enzyme activity from 0.239 ± 0.010 to 0.205 ± 0.001 U/mg biomass (*p* < 0.05). Rotation rate 120 rpm was chosen as the optimal rotation rate for production of glucose isomerase. a, b, c, and d indicate the significant difference between groups.

Rotation rate affects to dissolved oxygen concentration and nutrient diffusion in media. Therefore, the sufficient rotation rate will improve the bacterial growth and enzyme biosynthesis. However, too high dissolved oxygen concentration may cause a hyperbaric oxidative stress in prokaryotes, which in turn inhibits microbial growth and metabolism such as branched chain amino acid biosynthesis [18]. As shown in Figure 10, while the rotation rate increased from 80 to 120 rpm, the enzyme activity significantly increased (0.209 ± 0.004 and 0.248 ± 0.001 U/mg biomass, resp., *p* < 0.05) We observed that the faster rotation rate (140–180 rpm) reduced the enzyme activity from 0.239 ± 0.010 to 0.205 ± 0.001. U/mg biomass (*p* < 0.05). There is no difference of enzyme activity between rotation rates 120 rpm and 140 rpm. Therefore, the rotation rate 120 rpm was chosen as the optimal rotation rate for production of glucose isomerase to save energy. In conclusion, we found that the optimal fermentation conditions to produce glucose isomerase were pH 7.0, bacterial inoculation 20%, cultivation temperature 37°C, and rotation rate 120 rpm.

### 3.4. Screening of Significant Variables Using Plackett–Burman Design

For screening the significant variables for glucose isomerase production of *Bacillus megaterium*, impact levels of nine variables including medium components (D-xylose, K_2_HPO_4_, MgSO_4_·7H_2_O, yeast extract, and peptone) and fermentation conditions (pH, bacterial inoculum size, temperature, and rotation rate) were studied using the Plackett–Burman design for 12 runs and 9 two-level factors (−1 for a low level and +1 for a high level). In Plackett-Burman design, variables were encoded as follows: D-xylose concentration (*X*_1_), K_2_HPO_4_ concentration (*X*_2_), MgSO_4_·7H_2_O concentration (*X*_3_), yeast extract concentration (*X*_4_), peptone concentration (*X*_5_), initial pH (*X*_6_), bacterial inoculum size (*X*_7_), cultivation temperature (*X*_8_), and rotation rate (*X*_9_). Actual enzymatic activities of all variables with Plackett–Burman experimental design are presented in Table 1. The most prominent variables were determined by their actual enzymatic activities and their corresponding *p* values, and variables which have *p* values lower than 0.05 were considered to have statistical significant impact on glucose isomerase production. As shown in Table 2, three factors including D-xylose concentration (*X*_1_), yeast extract concentration (*X*_4_), and cultivation temperature (*X*_8_) have significant impact on glucose isomerase of *Bacillus megaterium*. Therefore, we selected these variables (D-xylose concentration, yeast extract concentration, and cultivation temperature) for further optimization experiments.

### 3.5. Optimization of Glucose Isomerase Production via Response Surface Methodology

The significant variables including D-xylose concentration (*X*_1_), yeast extract concentration (*X*_4_), and cultivation temperature (*X*_8_) were assessed at three coded levels (−1, 0, and +1) using response surface methodology with second-order polynomial model and a total 17 trials (Table 3). The general polynomial model equation was written as follows: *Y* = *a*_0_ + *a*_1_ *X*_1_ + *a*_2_ *X*_4_ + *a*_3_ *X*_8_ + *a*_12_ *X*_1_ *X*_4_ + *a*_23_ *X*_4_ *X*_8_ + *a*_13_ *X*_1_ *X*_8_ + *a*_11_ *X*_1_^2^ + *a*_22_ *X*_4_^2^ + *a*_33_ *X*_8_^2^, in which *Y* is the predicted enzyme activity, *X*_1_ is D-xylose concentration, *X*_4_ is yeast extract concentration, and *X*_8_ is cultivation temperature. The enzyme activity (*Y*) of each trial from experimental design was determined from replicated experiments and is presented as the average of replication in Table 4. The effect of each factors and the interaction of multiple factors on enzyme activity are presented in Table 5. Based on the full quadratic model application, it appeared that quadratic effect for yeast extract and cultivation temperature interaction (X_4_ *X*_8_) could be eliminated from the model because the coefficient for interaction was not significantly different with 0 (*p*=0.942). Therefore, polynomial model was rewritten as follows: *Y* = 0.277 + 0.006*X*_1_ + 0.012*X*_4_ − 0.004*X*_8_ + 0.004*X*_1_ *X*_4_ + 0.004*X*_1_ *X*_8_ − 0.016*X*_1_^2^ − 0.010*X*_4_^2^ − 0.007*X*_8_^2^. Based on this equation, we suggested that glucose isomerase activity (*Y*) was strongly affected by *X*_1_^2^, *X*_4_, *X*_4_^2^ (quadratic effect of D-xylose, yeast extract, and quadratic effect of yeast extract), whereas other variables had lower influence on glucose isomerase activity. Among variables, D-xylose concentration (*X*_1_), yeast extract concentration (*X*_4_), D-xylose concentration and yeast extract concentration interaction (*X*_1_·*X*_4_), and D-xylose concentration and cultivation temperature interaction (*X*_1_·*X*_8_) had positive effect on glucose isomerase activity, whereas other variables and interaction had negative effect on glucose isomerase activity.

Supplement with D-xylose for glucose isomerase production has been investigated in several microbes. Yassien and Jiman-Fatani suggested that supplement with 1% xylose in medium resulted in the maximal amount of glucose isomerase from *Streptomyces albaduncus* as compared to a variety of alternative carbon sources including glucose, lactose, maltose, mannitol, fructose, sucrose, inositol, galactose, and arabinose [17]. Furthermore, *Streptomyces thermonitrificans* could produce the highest glucose isomerase activity via cultivation in medium containing 1% xylose and 2% sorbitol [14]. Note that the improvement of xylose on glucose isomerase production is not related to bacterial growth. Some authors reported that xylose or combination of xylose and glucose decreased the biomass yield and increased enzyme activity [19, 20].

The importance of yeast extract on glucose isomerase production has been well described in several studies. According to Givry and Duchiron, the high amount of D-xylose isomerase produced by *Lactobacillus bifermentans* was only obtained in the presence of organic nitrogen sources, such as yeast extract, peptone, and meat extract, whereas the presence of inorganic nitrogen sources including ammonium citrate and ammonium phosphate did not have significant effect on D-xylose isomerase production [15]. In previous study, Nwokoro also suggested that yeast extract was the best nitrogen source for *B. licheniformis* to produce glucose isomerase among a variety of nitrogen sources including organic and inorganic sources [21]. Furthermore, Prabhakar and Raju proved that supplement with yeast extract reduced the dry weight of bacteria, but it increased glucose isomerase activity. This finding implied that yeast extract did not enhance *Arthrobacter* spp. growth, but it improved the effective utilization of medium components to produce glucose isomerase [9]. As a consequence, it is not surprising that yeast extract has the significant impact on glucose isomerase production of *B. megaterium*.

Each bacterium has a specific optimal range of temperature, in which it grows and synthesizes protein and secondary metabolic compounds. The optimal temperature for glucose isomerase was documented in several reports and was suggested in a wide range from 25-50°C. In previous study, Pandidurai et al. indicated that optimal cultivation temperature for production of glucose isomerase by *Enterobacter agglomerans* was 37°C with high glucose isomerase activity (41 U/mL), whereas the higher temperature resulted a decrease of enzyme activity [22]. On the other hand, *Penicillium fellutanum* produces the highest amount of glucose isomerase in 30°C [13]. Moreover, Habeeb et al. suggested the optimum temperature for *Streptomyces* spp. SH10 to produce glucose isomerase was 25°C [23]. Note that Nwokoro reported that *Bacillus licheniformis* produced the maximal amount of glucose isomerase at the higher temperature (50°C) [21]. Our results demonstrated the impact of cultivation temperature on glucose isomerase production and established the interaction between cultivation temperature and D-xylose on glucose isomerase production. The optimal value of cultivation temperature was also determined.

### 3.6. Model Validation

To reconfirm the regression equation, we performed variance analysis for parameters of response surface methodology fitted to equation for glucose isomerase production (Table 6). We found that *R* ^2^ value (coefficient of determination) was higher than 0.8 and close to 1.0 (*R* ^2^ = 0.971), and *Q* ^2^ value was higher than 0.5 (*Q* ^2^ = 0.794). Moreover, │*R* ^2^ − *Q* ^2^│value is lower than 0.2–0.3 and *p* value was lower than 0.05 (*p* < 0.001). These data satisfied all requirements for a good statistical model which could accurately predict data obtained from experiments [24]. Therefore, these data proved that this model is a good statistical model and the experimental data closely fitted with the model.

**Figure 11:**
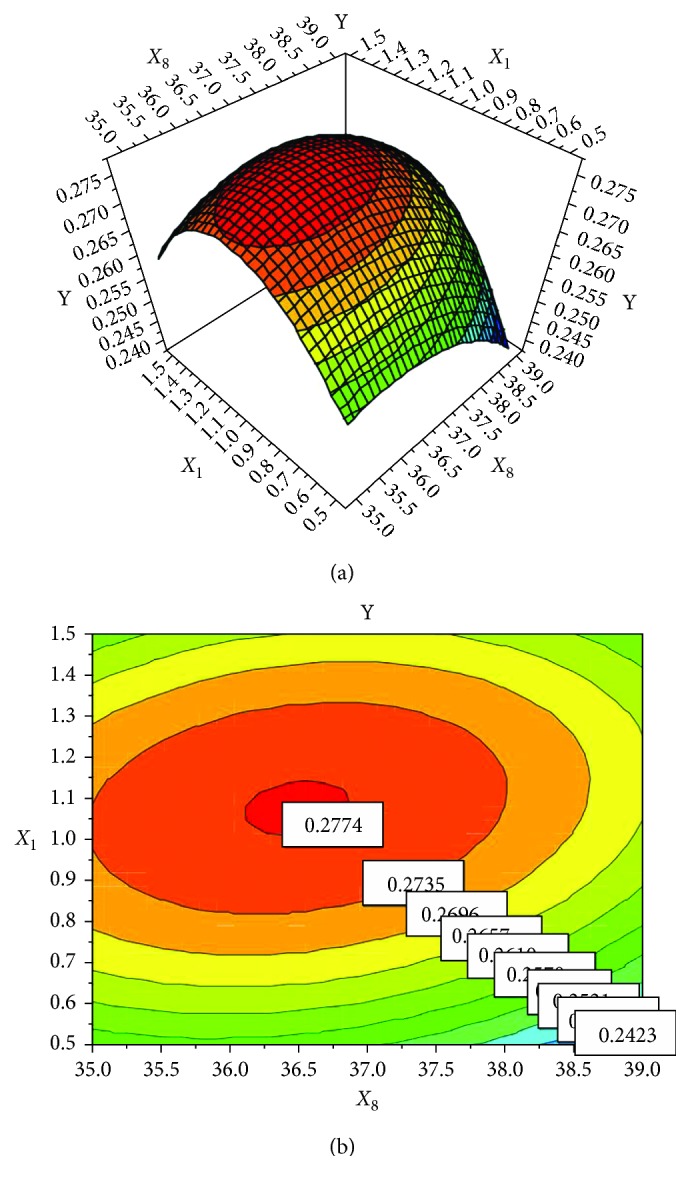
Response surface model and contour plots of the maximal glucose isomerase activity in the interaction of D-xylose concentration (*X*_1_) and cultivation temperature (*X*_8_) in the presence of yeast extract (1%).

The surface response models for optimization of glucose isomerase production showed that glucose isomerase production depended on interaction of D-xylose (*X*_1_), yeast extract (*X*_4_), and cultivation temperature (*X*_8_). The response surface and contour plot in Figure 11 show that optimal value of glucose isomerase activity (over 0.2774 U/mg biomass) obtained with the presence of D-xylose (1.0–1.2%) and yeast extract (1.1–1.2%) at cultivation temperature from 36 to 37°C.

Optimization experiments were conducted by Modde 5.0 software to determine optimal points of *X*_1_, *X*_4_, and *X*_8_. After that, we conducted the cultivation of *B. megaterium* with the optimal conditions obtained from Modde 5.0 software (D-xylose concentration = 1.116%, yeast extract concentration = 1.161%, and cultivation temperature = 36.528°C). The glucose isomerase activity that obtained from triplicate experiments (0.274 ± 0.003 U/mg biomass) was close to the predicted glucose isomerase activity obtained from the regression equation (0.278 U/mg biomass). The difference between experimental and predicted optimal values of enzyme activity was 1.439% (% difference <5%). Taken together, these data indicated that the statistical model was valid and acceptable for optimization of D-xylose, yeast extract, and cultivation temperature.

We also determined that the optimal conditions for glucose isomerase production of *B. megaterium* were medium containing D-xylose (1.116%), K_2_HPO_4_ (0.2%), MgSO_4_·7H_2_O (0.1%), yeast extract (1.161%), and peptone (1%) and specific fermentation conditions including initial pH 7, inoculum size (20% w/v), cultivation temperature (36.528°C), and incubation time (48 hours). The effective production of glucose isomerase was established from the statistical model and it could be applied in pilot or industrial scale production. Note that Mukesh-Kumar et al. reported the maximal glucose isomerase activity could be obtained via *B. megaterium* cultivation under the presence of D-xylose and peptone at 35°C, pH 6.0 for 48 hours [7]. The difference of isolated sources of *B. megaterium* may elucidate the difference of optimization conditions between our experiments and the previous study. Furthermore, the effects of medium components and fermentation conditions on glucose isomerase of other *Bacillus* strains were also well documented. Calik et al. suggested the optimal conditions for glucose isomerase production *Bacillus thermoantarcticus* as follows: birchwood xylan (1.06%), yeast extract (0.56%), (NH_4_)_2_SO_4_ (0.59%), pH 6.0, at 55°C. Moreover, Lawal et al. reported that the optimal pH and cultivation temperature to produce glucose isomerase from *Bacillus megaterium* and *Bacillus coagulans* were pH 5.0–9.0 and 30–70°C. Note that our optimization conditions were in agreement with optimal ranges of pH and cultivation temperature which reported in former research.

## 4. Conclusion

The influence of single factors and interaction of different factors on glucose isomerase production of *B. megaterium* were determined using Plackett–Burman design and response surface methodology. D-xylose, yeast extract, and temperature had strong impacts on enzyme activity. Note that D-xylose concentration, yeast extract concentration, and D-xylose and yeast extract interaction could significantly improve glucose isomerase activity. We successfully established optimal conditions for glucose isomerase production of *B. megaterium* as follows: D-xylose (1.116%), K_2_HPO_4_ (0.2%), MgSO_4_·7H_2_O (0.1%), yeast extract (1.161%), peptone (1%), pH 7.0, bacterial inoculum size (20% w/v), and cultivation temperature (36.528°C), for 48 hours with corresponding enzyme activity of 0.274 ± 0.003 U/mg biomass. These optimal conditions are considered useful in glucose isomerase production by *B. megaterium* in large scales with high cost-effectiveness.

## Figures and Tables

**Table 1:** Plackett–Burman experimental design matrix for screening variables having significant impact for glucose isomerase production by *Bacillus megaterium*.

Runs	Variables^*∗*^									Enzymatic activity (U/mg biomass)
	*X*_1_	*X*_2_	*X*_3_	*X*_4_	*X*_5_	*X*_6_	*X*_7_	*X*_8_	*X*_9_	
1	1.5	0.1	0.15	0.75	0.5	6.5	25	39	140	0.207
2	1.5	0.3	0.05	1.25	0.5	6.5	15	39	140	0.221
3	0.5	0.3	0.15	0.75	1.5	6.5	15	35	140	0.206
4	1.5	0.1	0.15	1.25	0.5	7.5	15	35	100	0.263
5	1.5	0.3	0.05	1.25	1.5	6.5	25	35	100	0.271
6	1.5	0.3	0.15	0.75	1.5	7.5	15	39	100	0.181
7	0.5	0.3	0.15	1.25	0.5	7.5	25	35	140	0.237
8	0.5	0.1	0.15	1.25	1.5	6.5	25	39	100	0.195
9	0.5	0.1	0.05	1.25	1.5	7.5	15	39	140	0.208
10	1.5	0.1	0.05	0.75	1.5	7.5	25	35	140	0.243
11	0.5	0.3	0.05	0.75	0.5	7.5	25	39	100	0.132
12	0.5	0.1	0.05	0.75	0.5	6.5	15	35	100	0.184

^*∗*^Variables are encoded as follows: D-xylose concentration (*X*_1_), K_2_HPO_4_ concentration (*X*_2_), MgSO_4_·7H_2_O concentration (*X*_3_), yeast extract concentration (*X*_4_), peptone concentration (*X*_5_), initial pH (*X*_6_), bacterial inoculum size (*X*_7_), cultivation temperature (*X*_8_), and rotation rate (*X*_9_).

**Table 2:** Experimental variables at different levels and their impacts on glucose isomerase production in Plackett–Burman experimental design matrix.

Factors		Levels		Impact levels	
Symbols	Variables	Low (−1)	High (+1)	Impact	Prob > *F* value
*X*_1_	D-xylose concentration (%)	0.5	1.5	0.019^b^	0.014
*X*_2_	K_2_HPO_4_ concentration (%)	0.1	0.3	−0.004^a^	0.194
*X*_3_	MgSO_4_ concentration (%)	0.05	0.15	0.002^a^	0.382
*X*_4_	Yeast extract concentration (%)	0.75	1.25	0.02^b^	0.012
*X*_5_	Peptone concentration (%)	0.5	1.5	0.005^a^	0.157
*X*_6_	pH	6.5	7.5	−0.002^a^	0.536
*X*_7_	Bacterial inoculum size (%)	15	25	0.002^a^	0.501
*X*_8_	Cultivation temperature (^o^C)	35	39	−0.002^b^	0.011
*X*_9_	Rotation rate (rpm)	100	140	0.008^a^	0.071

^*∗*^The letter “a” indicates no significant impact with 95% confidence level; the letter “b” indicates significant impact with 95% confidence level.

**Table 3:** Variables and their coded levels used for optimization of glucose isomerase production by *B. megaterium*.

Factors		Code levels		
Symbols	Variables	Low (−1)	Central (0)	High (+1)
*X*_1_	D-xylose concentration (%)	0.5	1	1.5
*X*_4_	Yeast extract concentration (%)	0.75	1	1.25
*X*_8_	Cultivation temperature (°C)	35	37	39

**Table 4:** Experimental design by RSM for D-xylose concentration, yeast extract concentration, and cultivation temperature to optimize glucose isomerase activity and experimental results.

Trials	Variables			Enzyme activity (U/mg biomass)
	*X*_1_	*X*_4_	*X*_8_	Experimental results
N1	0.5	0.75	35	0.238
N2	1.5	0.75	35	0.233
N3	0.5	1.25	35	0.257
N4	1.5	1.25	35	0.264
N5	0.5	0.75	39	0.222
N6	1.5	0.75	39	0.229
N7	0.5	1.25	39	0.236
N8	1.5	1.25	39	0.263
N9	0.5	1	37	0.247
N10	1.5	1	37	0.271
N11	1	0.75	37	0.253
N12	1	1.25	37	0.277
N13	1	1	35	0.269
N14	1	1	39	0.267
N15	1	1	37	0.279
N16	1	1	37	0.276
N17	1	1	37	0.280

**Table 5:** The impact of variables and their interaction on glucose isomerase activity.

*Y* (enzyme activity)	Coefficient values	Stand errors	*P* values
Constant	0.277	0.002	2.697 × 10^−13^
*X*_1_	0.006	0.001	0.004
*X*_4_	0.012	0.001	7.016 × 10^−5^
*X*_8_	−0.004	0.001	0.023
*X*_1_^2^	−0.016	0.003	0.001
*X*_4_^2^	−0.010	0.003	0.008
*X*_8_^2^	−0.007	0.003	0.036
*X*_1_·*X*_4_	0.004	0.002	0.041
*X*_1_·*X*_8_	0.004	0.002	0.041
*X*_4_·*X*_8_	−0.000	0.002	0.942

Variables are encoded as follows: D-xylose concentration (*X*_1_), yeast extract concentration (*X*_4_), and cultivation temperature (*X*_8_).

**Table 6:** Variance analysis for the parameters of response surface methodology fitted to second-order polynomial equation for glucose isomerase production.

Source of variance	Sum of squares	Degree of freedom	Mean of squares	*p*
Regression	0.006	9	0.001	*p* < 0.001
Residue error	0.000	7	2.178^–5^	—
Total	0.006	16	0.000	—
*R* ^2^	0.971			
*Q* ^2^	0.794			

## Data Availability

The dataset supporting the results of this article is included within the article and its supplementary materials.

## Supplementary Materials

Supplementary Materials Raw data that demonstrated bacterial growth and the effect of single factor including concentrations of D-xylose, K_2_HPO_4,_ MgSO_4_·7H_2_O, yeast extract, and peptone, as well as pH, bacterial inoculation size, temperature, rotation rate on glucose isomerase activity are available in excel sheets of supplementary files (Figures S1–S10).
